# Prevalence and determinants of antimicrobial resistance of pathogens isolated from cancer patients in an intensive care unit in Alexandria, Egypt

**DOI:** 10.1186/s42506-023-00134-8

**Published:** 2023-05-01

**Authors:** Nancy Mohamed, Abeer Ghazal, Asmaa Abdel Hameed Ahmed, Adel Zaki

**Affiliations:** 1 Department of Bioinformatics and Medical Statistics, Medical Research Institute, Alexandria University, Alexandria, Egypt; 2 Department of Microbiology, Medical Research Institute, Alexandria University, Alexandria, Egypt

**Keywords:** Multidrug resistance, Carbapenem resistance, Extended-spectrum beta-lactamase, Malignancy, Critical care

## Abstract

**Background:**

Infections caused by multidrug-resistant organisms (MDROs) are a globally increasing threat among critically ill patients, especially those with underlying malignancies. We aimed to assess the prevalence and susceptibility patterns of MDROs among cancer patients in intensive care units (ICU), and their predictors.

**Methods:**

Over 4 years, we retrospectively reviewed medical records of 497 malignancy patients in the ICU of a tertiary hospital in Alexandria, Egypt. The data for various factors, such as demographic characteristics, comorbidities, causative pathogen, and antimicrobial resistance (AMR), were collected and analyzed using univariate analysis. Logistic multivariate regression analysis was used to estimate the probability of developing MDROs among this population.

**Results:**

A total of 748 isolates were obtained from 1249 specimens. Gram-negative bacteria detected (459) comprised 61.4% of all isolates, while only 75 (10%) were gram-positive, and 214 (28.6%) were fungal pathogens. The most frequently encountered isolate was *Klebsiella pneumoniae* (*n* = 183), of which 107 were carbapenem-resistant (CR) and 62 were extended-spectrum beta-lactamase (ESBL)-producing. This was followed by *Escherichia coli* (*n* = 136), of which 17 were CR and 100 were ESBL-producing strains, while 3 were resistant to quinolones. *Acinetobacter baumannii* came in third (*n* = 67), with 63 being CR. The overall susceptibility of gram-negative bacteria was recorded as highest to colistin (97.3%). The prevalence of methicillin-resistant *Staphylococcus aureus* (MRSA) and Enterococcal species among gram-positive bacteria were 54.6% and 33.3%, respectively, with no resistance reported to vancomycin or linezolid. Among the MDRO infection predictors were neutropenia, recent antibiotics use, and receiving chemotherapy. Neutropenia had the highest odds ratio (OR: 2.3, CI: 1.28–4.09), followed by recent antibiotics use (OR: 1.8, CI: 1.22–2.59).

**Conclusion:**

Gram-negative bacilli were the most frequently reported MDROs, with resistance to higher generation cephalosporins and even carbapenems limiting antibiotic treatment options to older class antibiotics, such as colistin, with potential side effects, including nephrotoxicity. Estimating AMR probability using the prediction model of risk factors, such as neutropenia and previous antibiotics use, may be functional in the rapid identification of higher-risk patients.

## Introduction

Antibiotic resistance has been identified by the World Health Organization (WHO) as an international primary health concern [1]. Treating infection in critically ill patients is very challenging as multidrug-resistant (MDR) and pan-drug-resistant (PDR) pathogens are widely emerging in healthcare settings [2], resulting in antibiotic treatment failures, limited therapeutic choices, and altered effects of antibiotics. Multidrug-resistant organism (MDRO) infections delay clinical treatment and increase healthcare systems’ resource utilization [3]. The prevalence of infections among hospitalized patients is implicated by variable risk factors, such as accommodation in different hospital units—general wards versus intensive care units (ICU)—and altered individual’s immunity defense mechanisms caused by underlying comorbidities [4].

Patients with malignant tumors have weakened immune systems caused by tumor cell invasion and chemotherapy/radiotherapy [3]. Furthermore, previous antibiotics exposure, invasive operations, chemotherapy-induced mucositis, and previous hospitalizations increase the chance of infection by MDROs [5], resulting in delayed and reduced dosage of chemotherapeutics [6, 7].

In recent years, clinicians have also encountered infections due to MDR pathogens in oncology practice [8]. A rising trend of gram-negative bacilli (GNB) carrying extended-spectrum beta-lactamase (ESBL) genes and carbapenem resistance (CR) genes, as well as multidrug-resistant (MDR) gram-positive bacteria, methicillin-resistant *Staphylococcus aureus* (MRSA), and vancomycin-resistant Enterococci (VRE) were recently reported among this population [9].

Awareness of the local epidemiology of pathogens and their resistance patterns could improve the efficacy of treatment for cancer patients suffering from ICU infections [10]. The infectious disease pattern and sensitivity profiles differ widely from region to region, between units within a hospital, and even among the different ICUs within one hospital, arising from the selection pressure caused by antibiotic use [6].

The appropriate empiric antibiotic therapy is therefore critical to a successful outcome. Resistance patterns of bacterial isolates may determine empirical antimicrobials and positively influence patients’ prognosis [11].

Variable research on antimicrobial resistance (AMR) surveillance has been conducted; however, AMR monitoring studies among the critically ill population are scarce, specifically in developing countries, including Egypt [4].

This retrospective study aims to develop preliminary data on the frequency and clinical characteristics of the microbiological profile and explore the predictive factors of developing antibiotic resistance among samples of patients with malignant tumors admitted to the ICU of tertiary hospitals in Egypt.

## Methods

### Study design

A retrospective cohort study was carried out by reviewing medical records of patients admitted to the ICU of a tertiary care hospital in Alexandria, Egypt—a tertiary referral hospital with 50 ICU beds—over a period of 4 years, from January 2017 to December 2020, to identify the prevalence, determinants, and changes in antimicrobial resistance patterns among adult medical and surgical critically ill patients with malignancies.

### Inclusion criteria

By reviewing hospital medical records of ICUs, patients were included if:

- They were 18 years and older.
- They had a malignancy, confirmed by pathological and/or cytological tests.
- They had microbiological tests from suspected sites of infection, such as respiratory, urine, blood, aspirates, and swabs collected during their ICU stay.

If a patient had multiple admissions to a hospital ICU on more than one occasion, each admission was included separately in the analysis as an individual episode.

### Data collection

Through patient records and the microbiology laboratory database, including the patient’s demographics, indication for ICU admission, laboratory results, administration of corticosteroids and/or antibiotics within the last 90 days, Charlson Comorbidity Index (CCI) score—used to classify patients according to their comorbidities, such as diabetes, heart failure, immunity profile, chronic obstructive pulmonary disease, cerebrovascular stroke, chronic liver and kidney disease—[12, 13], length of stay, causative organism and infection site, and antibiogram.

### Operational definitions used throughout this study

Neutropenia: Absolute neutrophilic count (ANC) below 500 cells/mm^3^ or expected to decrease to less than 500 cells/mm^3^ during the next 48 h [14].

Adequate initial antibiotic therapy: Antimicrobial initiated was proven to have in vitro activity against the infecting strain according to antimicrobial susceptibility test results and the administration route and dosage were determined following current medical standards [15].

Prior antimicrobial therapy: Antimicrobial being administered within the last 3 months before the beginning of the infection episode [16, 17].

Multidrug-resistant bacterial infection (MDR): A gram-negative bacterium that is resistant to three different antibiotic groups. For gram-positive bacteria, methicillin resistance for *Staphylococcus aureus* (MRSA) and vancomycin resistance for *Enterococcus* species were considered MDR [18, 19].

Multidrug-resistant *Candida* infection: An isolate that is non-susceptible to ≥ 1 agent in ≥ 2 drug classes [20].

Septic shock: Sepsis accompanied by reduced organ perfusion and a need for a vasopressor administration to maintain blood pressure [21].

The Charlson Comorbidity Index (CCI) score: A list of 19 comorbid conditions, each having a weight assigned from 1 to 6 [22]. Scores of “1–2,” “3–4,” or “5 or more” are classified into “mild,” “moderate,” and “severe” illness, respectively. Moreover, survival rates over 5 years were 3.4%, 1.3%, and 1.3% in patients with CCI scores of mild, moderate, and severe, respectively [23, 24].

### Methods

During their ICU stay, variable microbiological specimens were collected from cancer patients with purulent respiratory secretions, febrile neutropenia—as neutrophils play a vital role in protecting against infection—symptomatic urinary tract infection, or any of the following alarming signs of infection: fever (temperature > 38 °C), chills, hypotension, or reduced organ perfusion; as well as patients with elevated inflammatory response indicators—e.g., C reactive protein (CRP), procalcitonin, leukocytosis, and leukopenia. Anomalies of the patient’s inflammatory response may signify a higher risk of serious illness.

### Interpretation of antimicrobial susceptibility testing results

Microbiological specimens were collected and cultured on blood, chocolate, and MacConkey agar. Additionally, blood samples were aerobically tested using the BACT/ALERT three-dimensional microbial detection system. Furthermore, bacterial identification and antimicrobial sensitivity tests were performed using the VITEK 2 compact automatic identification system. A modified Kirby Bauer disk diffusion method was performed to examine antibiotic susceptibility of ESBL gene-producing organisms and determine susceptibility to some of the antibiotics that were not included in the VITEK 2 antibiotic susceptibility testing (AST) panels.

Most of these approaches provide qualitative results—using the categories of susceptible, intermediate, or resistant—while some also yield quantitative data, given as the minimum inhibitory concentration (MIC) for each antibiotic—defined as the minimal antibiotic concentration that inhibits bacterial growth in a liquid medium.

For the cultures and AST, all the clinical samples were collected and analyzed. In the case of culture growth, the zones of antimicrobial inhibition were measured and interpreted according to the Clinical Laboratory Standard Institute (CLSI) 2017 breakpoints, to identify them as sensitive, intermediate, or resistant [25].

Isolates exhibiting the ESBL gene were identified by applying the double-disc synergy, looking for synergy between cephalosporin and clavulanic acid. ESBL production was detected if a ≥ eightfold reduction was observed in the MIC of a cephalosporin combined with clavulanic acid but not in that of cephalosporin alone [26]. Furthermore, CR for Enterobacteriaceae was considered with an MIC of > 8 for imipenem. Moreover, vancomycin resistance for *Enterococcus* species was considered with an MIC of > 4 mg/L [25].

### Statistical analysis

The statistical analysis was performed using the Statistical Package for Social Sciences (SPSS) version 20.0. Only the records that satisfied the inclusion criteria were used for data analysis. The descriptive statistics are presented as mean, standard deviation (SD), and median for the quantitative variables. Additionally, categorical variables are presented as frequency and proportions. The chi-square test (*χ*^2^) was used for categorical comparisons.

We examined the prognostic factors by univariate analyses; variables with a *P* value of < 0.05 in the univariate analysis were candidates for multivariate analysis. Logistic multivariate regression analysis was used to develop a clinical prediction model to estimate the probability of developing MDR among malignancy patients in the ICU.

Patient demographics, comorbidities, type of malignancy, malignancy treatment, hospital length of stay, and recent corticosteroids or antibiotic use were analyzed in relation to developing MDR.

All statistical tools were two-tailed and the level of significance was set at *p* < 0.05. The odds ratios (OR) and 95% confidence intervals (CI) were calculated to evaluate the strength of any association that emerged.

## Results

### Patient characteristics

Over the study period, 497 records of cancer patients in ICU were retrospectively analyzed.

Gastrointestinal tumors (42.5%) were the most common type of malignancy, followed by genitourinary tract tumors (20.7%) and pancreatic tumors (9.3%).

The malignancy was treated with chemotherapy in 51% of patients, and 17.7% were treated using radiotherapy, while combined chemo/radiotherapy was used in 15%. The patients’ demographics and characteristics are summarized in Table 1, which shows that 308 patients (62%) were male and 362 (72.8%) were aged above 60 years. Additionally, 245 patients (49.3%) had received prior antibiotics, while 83 (16.7%) received corticosteroids within the last 3 months before hospitalization, and 71 (14.8%) were neutropenic.

**Table 1:** Demographic characteristics, ICU admission indications, and relevant clinical data in 497 cancer patients, ICU in a tertiary care hospital, Alexandria, Egypt, 2017-2020

Patient characteristics	*N*	%
Demographics		
Male	308	62
Age > 60 years	362	72.8
Charlson Comorbidity Index score		
Mild	12	2.4
Moderate	49	9.9
Severe	436	87.7
Relevant clinical data		
Metastasis	188	27.8
Neutropenia	71	14.8
Antibiotics use within 3 months	245	49.3
Corticosteroids use within 3 months	83	16.7
Indications of ICU admission		
Sepsis	223	44.9
Shock	152	30.6
Others^a^	129	24.5
Duration of hospitalization in days	12.7 ± 9.2 (mean ± SD)	

^a ^Others: Acute kidney injury, end-stage liver disease, pleural effusion, hypoglycemia, road traffic accident

Moreover, the most frequent cause of ICU hospitalization among the patients with malignancy was sepsis (*n* = 223, 44.9%) followed by shock of variable causes, such as sepsis, hypovolemia, and cardiogenic shock (*n* = 152, 30.6%).

### Characteristics of causative pathogens

The culture reports of 1249 samples collected from medical and surgical ICU patients with malignancy were reviewed. Among the collected samples, the highest proportion of pathogens were isolated from urine cultures (34%), followed by respiratory cultures (27.8%) and blood cultures (23.8%). A total of 605 samples (48.4%) yielded no growth, while 540 (43.2%) yielded a single isolate, and 104 (8.4%) yielded multiple isolates. A total of 748 isolates were obtained from the collected clinical specimens (both single and multiple isolates); 459 (61.4%) were gram-negative bacteria, and only 75 (10%) were gram-positive bacteria, while 214 (28.6%) were fungal pathogens.

**Fig. 1:**
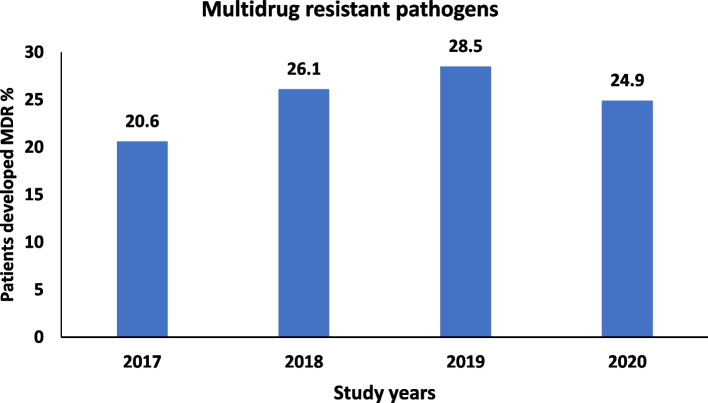
Drug resistance over different years within the study period (2017-2020), ICU in a turtiary care hospital, Alexandria, Egypt

As shown in Fig. 1, the highest percentage of patients who developed AMR (28.5%) was recorded in the third year of the study period, of which 84.9% were gram-negative MDROs. In the final study year, 24.9% of the pathogens were antibiotic-resistant, of which 88.9% were gram-negative MDR isolates. The difference in the frequency of patients who developed MDR pathogens over the study years was not statistically significant (*p* = 0.179).

### The antimicrobial sensitivity of organisms

We analyzed the AMR pattern under three categories, namely gram-negative, gram-positive, and fungal organisms. As shown in Table 2, the pathogen with the highest degree of resistance in addition to being the most frequent isolate—40% of all gram-negative bacteria episodes—was *Klebsiella pneumoniae* (*n* = 183), of which 107 were CR and 62 were ESBL strains. This was followed by *Escherichia coli* (*n* = 136), of which 17 were CR and 100 were ESBL strains, while 3 were quinolone-resistant. *Acinetobacter baumannii* came in third, with *n* = 67, of which 63 were CR.

**Table 2:** Percentage of antibiotic resistance of different organisms isolated from cancer patients in the ICU in a tertiary care hospital, Alexandria, Egypt, 2017-2020

All specimens results	MDR strains *n* (%)
**Gram-negative bacteria**	
*Klebsiella pneumoniae* (*n* = 183)	173 (94.5)
*Escherichia coli* (*n* = 136)	120 (88.25)
*Acinetobacter baumannii* (*n* = 67)	63 (94)
*Pseudomonas aeruginosa* (*n* = 30)	17 (56.7)
*Proteus mirabilis* (*n* = 20)	-
*Stenotrophomonas maltophilia* (*n* = 7)	7 (100)
*Enterobacter cloacae* (*n* = 5)	-
*Burkholderia cepacia* (*n* = 4)	-
Others^a^ (*n* = 7)	-
Total (*n* = 459)	380 (82.8)
**Gram-positive bacteria**	
*Staphylococcus aureus* (*n* = 41)	39 (95)
*Enterococcus faecalis* (*n* = 25)	25 (100)
*Enterococcus faecium* (*n* = 6)	6 (100)
*Streptococcus pneumoniae* (*n* = 2)	-
*Staphylococcus epidermidis* (*n* = 1)	-
Total (*n* = 75)	70 (93.3)
**Fungi**	
*Candida albicans* (*n* = 98)	-
*Candida tropicalis* (*n* = 72)	-
*Candida glabrata* (*n* = 24)	12 (50)
*Candida parapsilosis* (*n* = 11)	-
*Candida krusei*^b^ (*n* = 7)	7 (100)
*Cryptococcus laurentii* (*n* = 2)	-
Total (*n* = 214)	19 (8.9)

^a^Others: *Morganella morganii*, *Achromobacter xylosoxidans*, *Enterobacter asburiae*, *Citrobacter freundi*

^b^*Candida krusei*: Intrinsic resistance to fluconazole

Interestingly, among the gram-positive bacteria, *Staphylococcus aureus* was the most frequent isolate, representing 54.7% of all gram-positive episodes, of which 95% were MDR. However, no resistance to vancomycin, teicoplanin, or linezolid was encountered.

*Candida albicans* ranked third overall among the pathogens detected but first among the fungal isolates, representing 45.8% of all *Candida* isolates. No incidence of MDR strains was observed for *Candida albicans* (*n* = 98**)** nor *Candida tropicalis* (*n* = 72), while the resistance rate of *Candida glabrata* (*n* = 24**)** to fluconazole was above 50%.

*K. pneumoniae* was the most dominant among the respiratory isolates (30.6%), while *E.coli* was the dominant cause of urinary tract infection (38.2%) and MRSA (63.4%), and was the most dominant bacterial isolate obtained from blood samples.

Table 3 shows that the overall antibiotic susceptibility of GNB was recorded as highest to colistin (97.3%), followed by amikacin (63%), gentamicin (50.5%), then equally to imipenem and meropenem (37%). *E. coli*, *Acinetobacter baumannii*, and *Pseudomonas aeruginosa* were 100% colistin-susceptible.

**Table 3:**
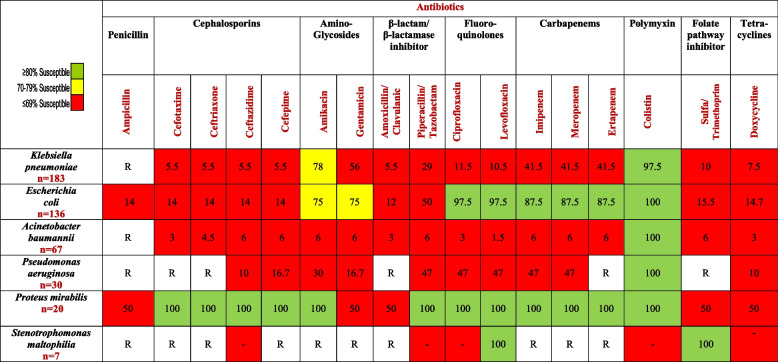
Percentage of antibiotic susceptibility pattern of predominant gram-negative bacterial isolates

Others: *Enterobacter cloacea, Burkholderia cepacian*, *Morganella morganii*, *Achromobacter xylosoxidans*, *Enterobacter asburiae*, *Citrobacter freundi*

*R*, intrinsic resistance

Resistance of *K. pneumoniae* to cephalosporins and β-lactam/β-lactamase inhibitor (BL-BLI) as piperacillin/tazobactam (Pip-Taz) ranged from 70 to 90%, while CR strains exceeded 50%. Similarly, the resistance rates of *E. coli* to cephalosporins were above 80%; in contrast, their aminoglycosides sensitivity reached 75%. Furthermore, the overall resistance of *Acinetobacter baumannii* to antimicrobials exceeded 90%.

Table 4 demonstrates that no resistance was encountered by *S. aureus* to vancomycin, linezolid, or teicoplanin. In contrast, the resistance rate of *S. aureus* to ampicillin reached 100% and was higher than 90% to trimethoprim/sulfamethoxazole. Similarly, no resistance was found for *Enterococcus* to vancomycin, linezolid, or teicoplanin; in contrast, its resistance rate to ampicillin was higher than 80%.

**Table 4:**
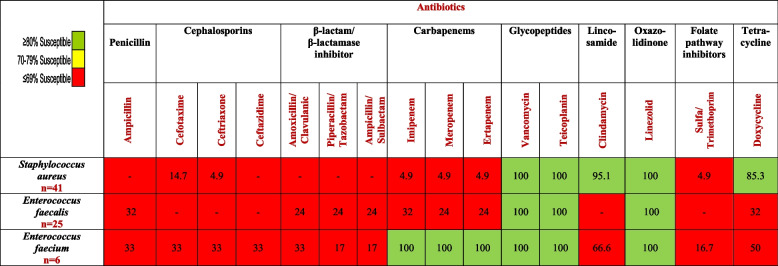
Percentage of antibiotic susceptibility pattern of predominant gram-positive bacterial isolates

Others: *Streptococcus pneumoniae*, *Staphylococcus epidermidis*

As shown in Table 5, the differences in frequencies of MDR isolates were statistically significant in patients with prolonged hospital stay (mean = 14.4 versus 11 days [*p* < 0.001]).

**Table 5:** Demographic and clinical predictors for development of MDR in ICU cancer patients (*n* = 497)—bivariate analysis

Patient comorbidities	MDR	Odds ratio	Confidence interval			*p*- value
			**Lower**		**Upper**	
**Age exceeding 60 years**						
**Yes** (*n* = 362)	186 (51.4%)	1.1	.72		1.59	0.728
**No** (*n* = 135)	67 (49.6%)					
**Charlson Comorbidity Index score**						
**Mild & Moderate** (*n* = 61)	37 (60.6%)	.643		.372	1.111	.111
**Severe** (*n* = 436)	217(49.7%)					
Duration of hospitalization*, (mean ± SD) days	253 (14.4 ± 11.07)	1.05		1.02	1.07	< .001*
Antibiotics use within 3 months *						
**Yes** (*n* = 245)	143 (58.4%)	1.8	1.25		2.5	< .001*
**N0** (*n* = 252)	110 (43.7%)					
**Corticosteroids within 3 months**						
**Yes** (*n* = 83)	48 (57.8%)	1.4	0.87		2.25	.167
**N0** (*n* = 414)	205 (49.5%)					
Neutropenic *						
**Yes** (*n* = 71)	51 (71.8%)	2.8	1.63		4.9	< .001*
**N0** (*n* = 426)	202 (47.4%)					
Chemotherapy *						
**Yes** (*n* = 253)	145 (57.3%)	1.7	1.19		2.41	< .001*
**No** (*n* = 244)	108 (44.3%)					
**Radiotherapy**						
**Yes** (*n* = 88)	41 (46.6%)	0.8	0.51		1.29	0.372
**No** (*n* = 409)	212 (51.8%)					
Metastasis *						
**Yes** (*n* = 188)	120 (63.8%)	2.4	1.64		3.47	< .001*
**No** (*n* = 309)	132 (42.7%)					

^*^Statistically significant

MDR isolates were more frequent in patients with metastasis (63.8% versus 36.2% [OR = 2.384]) and patients who received chemotherapy (57.3% versus 42.7% [OR = 1.691]). Furthermore, differences in MDR isolates were statistically significant in the patients with previous recent antimicrobial therapy group (58.4% versus 41.6% [OR = 1.81]) and neutropenic group (71.8% versus 28.2% [OR = 2.828]).

However, the clinical characteristics of these two groups demonstrated no significant differences between the two groups relative to age and CCI score. Also, bivariate analysis showed no significant differences between the two groups regarding their underlying type of malignancy (solid organ versus hematologic malignancy) and variable treatments.

As shown in Table 6, the multivariate analysis using a logistic regression model, including the variables associated with developing MDR by univariate analysis (*p* < 0.05), detected the significant independent risk factors for developing MDR as neutropenia, recent use of antibiotics, receiving chemotherapy, metastasis, and prolonged hospital stay. Neutropenia had the highest odds ratio (OR: 2.3, CI: 1.28–4.09), followed by recent antibiotics use (OR: 1.8, CI: 1.22–2.59).

**Table 6:** Adjusted effects of different studied risk factors for MDR in ICU cancer patients (*n* = 497)—multivariate analysis

Risk factors	Odds ratio	95% confidence interval		Sig
		**Lower**	**Upper**	
**Neutropenia**^a^	2.3	1.28	4.09	.005
**Antibiotics within 3 months**^a^	1.8	1.22	2.59	.003
**Received chemotherapy**^a^	1.5	1.01	2.18	.042
**Metastasis**	2.2	1.5	3.29	< .001*
**Duration of hospital stay in days**^a^	1.05	1.02	1.07	< .001*
**Constant**	0.236			

*Statistically significant

## Discussion

In this study, we aimed to identify the most prevalent pathogens among a large cohort of ICU patients with underlying malignancies, and the changes in the resistance patterns of these pathogens. The most impressive finding was that the most frequent isolates were GNB—459 isolates (61.2%). This finding was consistent with previous data from pediatric and adult critically ill patients at the Ain Shams University Hospitals in Egypt presented by Halim et al. [27] and Fahim et al. [26] respectively. These results were also supported by global findings in the recent years, wherein the isolates from cancer patients have shifted from gram-positive to gram-negative organisms [28].

Our results revealed that (1) the most commonly reported isolate—39.9% of all gram-negative isolates—was *K. pneumoniae* (*n* = 183); (2) the majority of this organism—173 isolates (94.5%)—were MDR; (3) the second most frequently isolated organism—29.6% of all gram-negative isolates—was *E. coli* (*n* = 136); and (4) of which 120 isolates (88.25%) were MDR.

A similar trend was also reported locally in Egypt by Khalifa et al. [29], as well as globally among patients with underlying malignancies in tertiary hospitals by Paul et al. [30] and Garg et al. [6]. This was in contrast to the study carried out by Rezk et al. in a critical care unit in Egypt that reported *Acinetobacter baumannii* as the predominant organism [31]. Moreover, Sheth et al. found that among ICU patients, *Acinetobacter* spp. and *Pseudomonas* spp. were the major causes of infections [32].

Our cumulative data disclosed antibiotic resistance among *K. pneumoniae* and *E. coli* spp., reaching 80–90%, to third generation cephalosporins (ceftriaxone/ceftazidime), fourth generation cephalosporins (cefepime), and BL-BLI combinations, such as ampicillin/sulbactam and Pip-Taz. Similar high rates of resistance of these organisms to third generation cephalosporins have been noted locally by Halim et al. in a study in Egypt [27] as well as Southern India [4].

Distinct from data gathered by Kapoor et al. [11] from an oncology center in India and by Cattaneo et al. [33] from hematological institutions in Italy, no VRE were recorded in our study. Similarly, no *S. aureus* resistance to vancomycin, linezolid, or glycopeptides was observed, contrary to data collected from a university hospital in Egypt by Zahran et al. [34] and by Shebl et al. [35], wherein vancomycin resistance reached 9.5% and linezolid resistance 10.8%.

In contrast to a study carried out at a cancer teaching center in China [36], *Candida albicans* strains expressed no resistance to fluconazole in our study.

Our results showed that the highest frequency of pathogens was found in the urine cultures followed by respiratory cultures. These results differ from the findings in a study carried out in the ICU of the Ain Shams Hospital in Egypt that reported the highest proportion of pathogens from blood cultures [26].

Owing to the increase in prevalence of antibiotic-resistant organisms, it was important to examine the factors that may contribute to the globally rising prevalence of antibiotic resistance. In our study findings, the significant predictors for development of MDR included neutropenia, antibiotics use within the last 3 months, chemotherapy, metastasis, and prolonged hospital stay.

Similarly, another study found that the main contributing factors to the development and spread of antibiotic-resistant infections in critical care units include the prolonged use of antimicrobials as well as the presence of fragile geriatric patients, immunosuppressed patients that are highly susceptible to acquiring MDR infections—e.g., AIDS patients, transplant recipients, and cancer patients—undergoing invasive surgical procedures, and prolonged length of stay in hospital [37, 38].

### Limitations and strength

We are aware of the limitations of this study, having been carried out retrospectively and the data collected from patients’ records. This does not significantly affect the validity of our results as we revised the accuracy and completeness of data in the records and found them satisfactory.

Additionally, as our study was conducted at one hospital, the results may not be representative for all hospitals in Alexandria. However, owing to the large sample size and the study having been done in the largest tertiary hospital in the city with the highest level of healthcare facilities for cancer patients, it could be reasonably representative of ICU cancer patients in our community.

## Conclusion

Among ICU patients with malignancies, the incidence of MDR-GNB resistant to higher generation cephalosporins and even carbapenems was rising over the study years, limiting antibiotic treatment options to only older classes of antibiotics, such as colistin and aminoglycosides, with potential side effects, including nephrotoxicity. Estimating AMR probability using the clinical prediction model of risk factors, such as neutropenia and previous antibiotics use, may be functional in stratifying higher risk patients. This study may provide valuable data for national surveillance of MDR and comparisons with other countries. This could be a steppingstone to generating a robust local antibiotic policy.

## Data Availability

The datasets analyzed during the current study are not publicly available due to patient’s privacy but are available from the corresponding author upon reasonable request.

## Abbreviations

AMR: Antimicrobial resistance
ANC: Absolute neutrophil count
CRP: C-reactive protein
CCI: Charlson Comorbidity Index
CI: Confidence intervals
CR: Carbapenem resistant
ESBL: Extended spectrum beta lactamase
GNB: Gram-negative bacilli
ICU: Intensive care units
MDRO: Multidrug-resistant organisms
MRSA: Methicillin-resistant Staphylococcus aureus
OR: Odds ratios
SD: Standard deviation
VRE: Vancomycin-resistant *Enterococcus*
